# Social factors of health-related quality of life in older adults: a multivariable analysis

**DOI:** 10.1007/s11136-023-03472-4

**Published:** 2023-07-17

**Authors:** Christoph Geigl, Julika Loss, Michael Leitzmann, Christian Janssen

**Affiliations:** 1 Department of Applied Social Sciences, Munich University of Applied Sciences, 81243 Munich, Germany; 2 Department of Epidemiology and Preventive Medicine, University of Regensburg, 93053 Regensburg, Germany; 3 Department of Epidemiology and Health Monitoring, Robert Koch Institute, 13353 Berlin, Germany

**Keywords:** Health-related quality of life, HRQOL, SF-36, Social factors, Older German adults, Multiple linear regression

## Abstract

**Purpose:**

The objective of the analysis was to examine the relationships between sociodemographic, socioeconomic, psychosocial, and behavioural factors and both physical and mental health-related quality of life (HRQOL) in older adults.

**Methods:**

The analysis was based on recent cross-sectional data of 1687 community residents from a whole population postal survey of German adults aged 65 years and older (33% response rate, 52% female, mean age 76 years). HRQOL was assessed using the 36-Item Short Form Survey (SF-36v2). For a differentiated analysis, hierarchical multiple linear regressions were performed.

**Results:**

An internal health locus of control, physical activity, social support, and income were positively associated with physical HRQOL (Adj. *R*^2^ = 0.34; *p* < 0.001) and mental HRQOL (Adj. *R*^2^ = 0.18; *p* < 0.001), whereas an external health locus of control and age were negatively associated with both. Alcohol use and educational level were positively associated only with physical HRQOL, whilst female gender was negatively associated only with mental HRQOL.

**Conclusion:**

Sociodemographic, socioeconomic, psychosocial, and behavioural factors were associated with physical and mental HRQOL. These results highlight the importance of social factors in HRQOL and provide approaches for policy and practice to develop and implement tailored health interventions for older adults. Our findings may be transferable to municipalities in metropolitan areas of high-income European countries.

**Clinical trial registration:** Not applicable.

## Plain English summary

Most relationships between social factors and health-related quality of life in older adults have not yet been established for the general older population. Understanding the relationships between social factors and health-related quality of life using recent data from an older adult population may provide important new insights for quality of life research in this specific age group. We aimed to investigate the associations between social factors and both physical and mental health-related quality of life in older German adults. Our findings can help to understand older adults’ needs, adequately address age-specific health interventions, and reduce health inequities amongst older adults. We demonstrate that sociodemographic, socioeconomic, psychosocial, and behavioural factors are associated with physical and mental health-related quality of life. Overall, our results indicate that social factors should be considered when targeting interventions to reduce health inequity in older age. Depending on the focus of the intervention, it may be appropriate to take certain social conditions into account to maintain or improve the health-related quality of life of older adults.

## Background

Health-related quality of life (HRQOL) is a multidimensional concept of health that refers to functioning and perceived well-being in the physical, mental, and social domains of life [1]. HRQOL is considered an essential indicator of older adults’ overall health status [2, 3] and is highly relevant for assessing independent living in older age [4]. From a societal perspective, it is necessary to assess older adults’ health beyond the increasing prevalence of multimorbid chronic diseases [5–8] and disabilities [9, 10] as these are not necessarily decisive factors for rating older adults’ HRQOL [11, 12]. Regardless of objective health status, self-rated HRQOL is a robust predictor of mortality amongst older adults [13–15]. Recent studies amongst older German adults suggest that the HRQOL of older adults deteriorated with the COVID-19 pandemic. At the same time, many older adults appear to possess coping resources that enable them to deal with the consequences of the pandemic better than younger age groups [16–18]. Older adults’ HRQOL has grown in importance with rapidly ageing populations worldwide [19]. In the European Union (EU), the population aged 65 and older will increase from 90.5 million older adults at the beginning of 2019 to 129.8 million in 2050. At the same time, the number of people aged 75–84 is expected to increase by 56% [20]. Germany is one of the EU countries most affected by population ageing [10]. In 2023, the share of the population aged 65 years and older amounted to 23 percent (19 million people) with an upwards trend, meaning that almost one in four Germans is aged 65 years or above [21].

In ageing populations, the desire to spend the most advanced years in a good self-assessed health condition has gained more relevance [3]. To cope with the challenges arising from this trend, most older adults prefer to remain in their familiar environment in the community and live with some level of independence [22–25]. Thus, there is growing interest in understanding the relationships between HRQOL and its associated factors in older adult populations. It is well known that chronic diseases and multi-morbidity are associated with worse HRQOL in older adults [26–28]. Both physical limitations, such as impaired mobility or reduced physical function [29, 30], and mental diseases, such as depression or anxiety disorder [31, 32], have been shown to be associated with worse HRQOL in older adults. Recently, environmental conditions such as satisfaction with the living environment and health services have also been identified as relevant factors associated with higher HRQOL amongst older adults [33]. In addition to physical, mental, and environmental aspects, social factors may play an important role in HRQOL [34]. However, only a few population-based studies have examined the relationships between social factors and HRQOL in older adults. Previous studies have reported that older age [35–37], female gender [35, 37], lower education [37, 38], lower occupational status [39, 40], lower income [41, 42], an external health locus of control [43, 44], less social support [45, 46], and less physical activity [47, 48] are associated with lower HRQOL. With regard to partnership status, contrasting results have been found. Studies of older adults have found both a negative association between older adults living in a partnership [49] and those living alone without a partner [50].

Concerning smoking status, there are also contradictory results. Studies have shown that older adult smokers indicate worse quality of life [51] and that current smokers have the highest HRQOL compared to never smokers and former smokers [52]. In the case of alcohol consumption, the associations with HRQOL also appear to be more complex. In a prospective study, positive cross-sectional associations but no prospective associations between alcohol consumption and HRQOL were found [53], whereas another longitudinal study found that persistent moderate drinkers had the highest HRQOL compared to all other groups [54]. Aside from gender- and age-related HRQOL associations, most of the relationships between social factors and HRQOL have not yet been examined in older general populations. Thus, the results are often limited to samples of older adults with specific diseases, and in many analyses, social factors are merely used as confounders. Moreover, we are not aware of any recent population-based studies that have examined the associations between sociodemographic, socioeconomic, psychosocial, and behavioural factors and both physical and mental HRQOL amongst older German adults in comprehensive statistical models.

A differentiated understanding of the structural relationships between social factors and HRQOL based on a recent sample of older German adults may provide important new insights for HRQOL research in this specific age group. Reflecting established theoretical models [55–57], we conceptualise social factors as a multidimensional construct that includes sociodemographic, socioeconomic, psychosocial, and behavioural determinants of health inequalities. The findings of this work could serve to identify groups with social risk profiles that might particularly benefit from interventions to maintain and improve physical and mental HRQOL in older adults. Furthermore, considering social factors when targeting interventions may help to reduce socially determined health inequities amongst older German adults. The objective of the analysis are to examine the

1. associations between sociodemographic, socioeconomic, psychosocial, and behavioural factors and both physical and mental HRQOL in older adults.

## Methods

### Data and sample

**Fig. 1:**
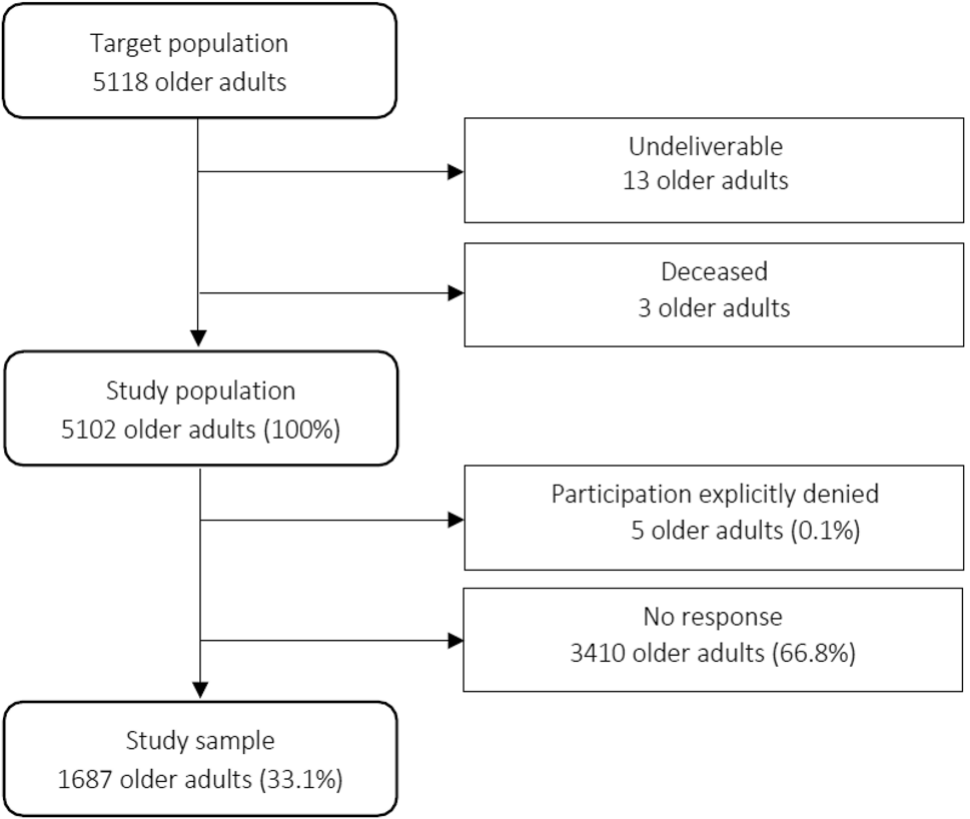
Participant flow diagram

The analysis was based on cross-sectional data gathered as part of the healthy municipality project conducted in Puchheim, Germany (*n* = 1687, 33% response proportion, 52% female, mean age = 76 years). From May to August 2019, a whole population postal survey was conducted to collect actual health-related data from adults aged 65 and older. The address data of 5102 older adults were provided by the municipal administration and the residents’ registration office. All eligible residents received a written standardised self-administered questionnaire (SAQ) along with a letter from the principal mayor and the project manager. A total of 1687 older adults answered and returned the questionnaire (Fig. 1).

### Measures

HRQOL was assessed using the 36-Item Short Form Survey (SF-36) [58], version 2 [59]. This reliable and validated instrument has been successfully applied in population-based surveys in Germany [60–62] as well as with older adult populations [63–65]. The SF-36 includes the eight subscales of physical functioning, role physical, bodily pain, general health, vitality, social functioning, role emotional, and mental health (Cronbach’s alpha ranged from 0.78 to 0.95). Based on these subscales, a physical component summary (PCS; physical HRQOL) and a mental component summary (MCS, mental HRQOL) were calculated. The component summaries were scored by weighting and summing the eight subscales. The data were adequate for principal component analysis (PCA) to analyse the dimensional structure: The Kaiser–Meyer–Olkin measure of sampling adequacy (MSA) was 0.9 (marvellous) [66], the *p*-value of Bartlett’s test for sphericity was significant (*p* < 0.001), and the anti-image correlation values ranged from 0.85 to 0.96. The postulated two-dimensional structure of the HRQOL could be confirmed in the PCA. Despite the expected mixed component loadings, communalities (> 0.6) and component loadings (> 0.7) fulfilled the statistical requirements for an appropriate representation of the underlying two-dimensional construct [67]. The two principal components, physical and mental HRQOL, represented 74.3% of the total variance of the eight subscales. Both summary measures were calculated using American weights. Finally, the scores were standardised to a mean of 50 and a standard deviation of 10. Higher values represented higher physical and mental HRQOL. Socioeconomic status (SES) variables were calculated as predefined point scores of educational level, (previous) occupational status, and equivalised disposable income [68–70]. Higher values indicated higher educational levels, higher occupational status, and higher incomes. A multidimensional Health Locus of Control Scale (HLC) [71] was used to assess the level of belief that health and illness are internally or externally controlled. Higher values corresponded to more pronounced internal or external control tendencies. Social support was measured using the Oslo 3-Item Social Support Scale (OSS-3) [72], with higher values reflecting stronger levels of social support [73]. To assess alcohol use, the Alcohol Use Disorder Identification Test-Consumption (AUDIT-C) [74] was utilised. Higher values represented higher alcohol consumption. Physical activity (PA) [75] was assessed by the frequencies of sport and more physically demanding daily activities, with higher values indicating higher physical activity. Smoking status [76] was presented as a dichotomous risk behaviour (current smoker/non-smoker). Age was calculated based on the year of birth. Recognising that the diversity of gender identities cannot be captured with binary response options (male/female) [77], we used the term “gender” to emphasise socially constructed differences that go beyond biological sex [78]. Partnership status indicated whether respondents lived in a partnership or not (no partner/having a partner). The required information was collected according to national demographic standards [79]. A more detailed description of the measurement instruments, the operationalisation, and the psychometric proprieties of the social factor variables are provided in another work by the authors [80].

### Statistical analysis

Sample characteristics were given as absolute and relative frequencies of valid and missing values, mean, standard deviation, minimum, and maximum for sociodemographic, socioeconomic, psychosocial, and behavioural variables as well as for physical and mental HRQOL. To examine missing data, *t tests* were used to analyse the mean differences between valid and missing values concerning sociodemographic and socioeconomic standard variables.

Missing values of the variables used in the correlation and regression analysis were estimated by multiple imputation (MI) [81]. Compared to other methods for dealing with missing data, MI provides relatively precise estimations regardless of the missing mechanism [82]. Inappropriate handling of missing values may weaken the validity of results and conclusions [83, 84]. All analysis variables and auxiliary variables (SF-36 subscales, size, weight) were included in the imputation model. The number of imputation data sets to be calculated was set at five (*m* = 5) on theoretical grounds. Thus, the output data set consisted of the original case data and five complete data sets with imputed values. Predictive mean matching (PMM) was utilised as the imputation method (*k* = 5), the only method to date that provides plausible imputations and preserves the original data distributions [85]. The results of the analysis were generated for the original data set, each of the five complete data sets, and a pooled output. We routinely conducted sensitivity analysis to compare results between the primary multiple imputed data analysis and the corresponding complete case analysis (list-wise deletion) [86].

Multiple linear regressions were performed to analyse the associations between social factors and both physical and mental HRQOL. Comprehensive statistical models were examined separately for physical and mental HRQOL. Using a hierarchical method, social factors were introduced according to a predefined theoretical model structure. Standardised regression coefficients (*β*) were employed to interpret the strength and direction of the associations between each social factor and HRQOL. At the model level, coefficients of determination (*R*^2^) were utilised to interpret the strength of the association between introduced social factors and HRQOL. Adjusted coefficients of determination (Adj. *R*^2^) were interpreted as goodness-of-fit measures (model accuracy) following Cohen [87]. The reported *R*^2^ and *F* values were averaged across all imputed data sets (pooled) [88, 89]. The *F* change (∆*F)* was used to assess whether the blocks of variables introduced significantly improved the goodness of fit of the models (∆*R*^2^). All assumptions (linearity, multicollinearity, homoscedasticity, independence, normality) for linear regression analysis were carefully checked and considered to be met [67]. Heteroscedasticity-consistent standard error estimators (HC4) were used by default to ensure validity and power [90]. Statistical analysis was conducted with IBM® SPSS® Statistics software (version 27.0.0.0). Statistical significance levels were defined as *p* < 0.05.

## Results

### Univariate analysis of sample characteristics

Table 1 shows the characteristics of the total sample. The sample comprised 791 males (47.6%) and 870 females with an average age of 76.4 years (range 65–98 years). Whilst 1116 respondents (68.3%) were aged 65–79 years, 519 respondents were aged 80 years or older. The nationality of the broad majority (97.2%) was German. Respondents who did not provide sufficient information to calculate SF-36 component summaries and AUDIT-C scores were older (*p* < 0.001) and had a lower socioeconomic status (*p* < 0.001) on average.

**Table 1:** Descriptive statistics of the sample

Variables	Valid values *n*	Missing values *n* (%)	Mn	Mx	M	SD
*Sociodemographic factors*						
Age in years	1635	52 (3.1)	65	98	76.4	6.3
Gender^b^	1661	26 (1.5)	0	1	0.52^a^	0.5
Partnership status^c^	1661	26 (1.5)	0	1	0.72^a^	0.5
*Socioeconomic factors*						
Educational level	1666	21 (1.2)	1	7	4.1	1.6
Occupational status	1650	37 (2.2)	1	7	4.8	1.2
Income	1593	94 (5.6)	1	7	4.0	1.9
*Psychosocial factors*						
Internal health locus of control	1633	54 (3.2)	1	5	2.9	0.7
External health locus of control	1642	45 (2.7)	1	5	2.7	0.7
Social support	1647	40 (2.4)	3	14	9.7	2.0
*Behavioural factors*						
Smoking^d^	1674	13 (0.8)	0	1	0.06^a^	0.2
Alcohol use	1516	171 (10.1)	0	11	2.9	1.9
Physical activity	1628	59 (3.5)	2	12	8.2	2.4
*SF-36 component summaries*						
Physical health-related quality of life	1528	159 (9.4)	6	66	43.9	10.3
Mental health-related quality of life	1528	159 (9.4)	4	70	50.0	9.7

*n*: valid values; Mn: minimum; Mx: maximum; *M*: mean; *SD*: standard deviation

^a^The mean can be interpreted as a percentage of the distribution

^b^The reference group is male

^c^The reference group is no partner

^d^The reference group is non-smokers

### Multivariable relationships between social factors and health-related quality of life

The results of the linear regression between social factors and physical HRQOL are shown in Table 2. In the final model, internal HLC, physical activity, income, alcohol use, educational level, and social support were positively associated with physical HRQOL, whilst age and external HLC were negatively associated. Each introduced variable block improved the model’s goodness of fit (*p* < 0.001). The final model indicated high goodness of fit [87]. In the corresponding complete case analysis, educational level (*β* = 0.04; *p* = 0.19) and social support (*β* = 0.03; *p* = 0.28) were not significantly associated.

**Table 2:** Multiple linear regression between social factors and physical health-related quality of life

Model	M 1		M 2		M 3		M 4	
	*β*	p	*β*	*p*	*β*	*p*	*β*	*p*
*Sociodemographic factors*								
Age	− 0.36	< 0.001	− 0.34	< 0.001	− 0.28	< 0.001	− 0.25	< 0.001
Gender^a^	− 0.07	0.007	− 0.02	0.33	0.01	0.84	0.01	0.57
Partnership status^b^	0.05	0.046	0.05	0.07	0.06	0.02	0.03	0.15
*Socioeconomic factors*								
Educational level			0.09	0.001	0.08	0.02	0.06	0.01
Occupational status			0.03	0.34	0.00	0.97	− 0.01	0.69
Income			0.08	0.003	0.08	0.001	0.07	0.004
*Psychosocial factors*								
Internal health locus of control					0.32	< 0.001	0.29	< 0.001
External health locus of control					− 0.19	< 0.001	− 0.14	< 0.001
Social support					0.07	0.001	0.06	0.01
*Behavioural factors*								
Smoking^c^							0.02	0.43
Alcohol use							0.08	0.001
Physical activity							0.24	< 0.001
*R*^2^	0.14		0.16		0.29		0.34	
Adj. *R*^2^	0.14		0.16		0.28		0.34	
Δ *R*^2^	0.14		0.02		0.13		0.05	
*F*	93.83	< 0.001	54.42	< 0.001	73.46	< 0.001	72.58	< 0.001

*n* = 1687; *β*: standardised regression coefficient;* p*: *p value*. *R*^2^: coefficient of determination (pooled); Adj. *R*^2^: adjusted coefficient of determination (pooled); Δ *R*^2^: coefficient of determination change (pooled); *F*: *F*-statistic (pooled). Robust standard errors are between 0.02 and 0.03 for all coefficients

^a^The reference group is male

^b^The reference group is no partner

^c^The reference group is non-smokers

The results of the linear regression between social factors and mental HRQOL are shown in Table 3. In the final model, social support, internal HLC, physical activity, and income were positively associated with mental HRQOL, whilst external HLC, age, and female gender were negatively associated. Each introduced variable block improved the model’s goodness of fit (*p* < 0.001). The final model indicated moderate goodness of fit [87].

**Table 3:** Multiple linear regression between social factors and mental health-related quality of life

Model	M 1		M 2		M 3		M 4	
	*β*	*p*	*β*	*p*	*β*	*p*	*β*	*p*
*Sociodemographic factors*								
Age	− 0.16	< 0.001	− 0.14	< 0.001	− 0.09	< 0.001	− 0.09	0.001
Gender ^a^	− 0.11	< 0.001	− 0.07	0.01	− 0.06	0.02	− 0.06	0.02
Partnership status ^b^	0.06	0.02	0.05	0.06	0.04	0.15	0.03	0.29
*Socioeconomic factors*								
Educational level			0.06	0.04	0.04	0.14	0.03	0.23
Occupational status			0.05	0.09	0.03	0.28	0.02	0.39
Income			0.14	< 0.001	0.11	< 0.001	0.11	< 0.001
*Psychosocial factors*								
Internal health locus of control					0.14	< 0.001	0.13	< 0.001
External health locus of control					− 0.15	< 0.001	− 0.13	< 0.001
Social support					0.24	< 0.001	0.23	< 0.001
*Behavioural factors*								
Smoking ^c^							− 0.02	0.38
Alcohol use							0.01	0.73
Physical activity							0.11	< 0.001
*R*^*2*^	0.05		0.08		0.17		0.18	
Adj. *R*^2^	0.05		0.08		0.17		0.18	
Δ *R*^2^	0.05		0.03		0.09		0.01	
*F*	27.48	< 0.001	23.91	< 0.001	38.60	< 0.001	31.39	< 0.001

*n* = 1687; *β*: standardised regression coefficient;* p*: *p value*. *R*^2^: Coefficient of determination (pooled); Adj. *R*^2^: adjusted coefficient of determination (pooled); Δ *R*^2^: coefficient of determination change (pooled); *F*: *F*-statistic (pooled). Robust standard errors are between 0.02 and 0.03 for all coefficients

^a^The reference group is male

^b^The reference group is no partner

^c^The reference group is non-smokers

## Discussion

### Principal findings

Sociodemographic, socioeconomic, psychosocial, and behavioural factors were significantly associated with physical and mental HRQOL. Internal HLC, physical activity, social support, and income were positively associated with both physical and mental HRQOL, whereas external HLC and age were negatively associated with both. Alcohol use and educational level were positively associated only with physical HRQOL, whilst female gender was negatively associated only with mental HRQOL. The explained variance of physical HRQOL was strong, and the explained variance of mental HRQOL was moderate to strong [87], highlighting the importance of social factors for older adults’ HRQOL. The relevance of HLC for physical and mental HRQOL was somewhat surprising. These findings are a valuable contribution to HRQOL research in older adults as they provide promising approaches to maintaining or increasing HRQOL in this specific age group.

### Comparison with other studies

Age was negatively associated with physical and mental HRQOL, whereas previous studies [35–37] have indicated this association only for physical HRQOL. Surprisingly, we were able to demonstrate age effects on mental HRQOL. Although other studies [35–37] found negative associations of female gender with physical and mental HRQOL, we found a negative association only with mental HRQOL. However, gender may moderate the relationships between social factors and HRQOL [36]. Further research should investigate the moderating effects of gender by estimating corresponding interaction effects. With regard to partnership status, previous studies discovered a negative association between older adults living in a partnership [49] and those living alone without a partner [50]. Even though we did not find significant associations between partnership status and HRQOL, future studies should consider potential partnership status differences in their analyses.

In line with previous studies, educational level was positively associated only with physical HRQOL [37, 38], whilst income was positively associated with physical and mental HRQOL [41, 42]. However, in contrast to other studies [39, 40], we did not find associations between occupational level and HRQOL. In the acquisition of the data, occupational status was limited to upper categories. Therefore, linear relationships could have remained hidden due to the low level of differentiation. In summary, income was the main socioeconomic factor in our analysis. This finding is a relevant addition to the field of HRQOL research in older adults that should be confirmed by further research.

The relationship between HLC and HRQOL in older adults has rarely been studied. However, our findings are consistent with those of previous studies revealing that internal HLC is positively associated with HRQOL in older adults [44], whereas external HLC is negatively associated with HRQOL [43, 44]. In the original concept by Wallston et al. [91], health locus of control beliefs are not considered as stable as more general control beliefs. Therefore, health-related control beliefs should not be seen as an indicator of a personality trait but rather as a disposition to behave in a certain way in health-related situations. This disposition may change with new experiences or changes in the situation. Thus, associations in cross-sectional designs should consider the possibility that the HLC may be partly determined by health status. This is also consistent with Rotter’s [92] social learning theory, which holds that one’s expectations are the result of past experiences. Overall, the processes are assumed to be reciprocal and cyclical, as health outcomes and experiences (e.g. memory or physical deterioration) may have an impact on control beliefs, which in turn can affect behavioural factors and future health outcomes [93]. In further studies, the role of HLC should be investigated more deeply with more differentiated scales, such as a four-dimensional (self-control, self-blame, powerful others, chance) scale. Social support was positively associated with physical and mental HRQOL. This is in line with a longitudinal population-based study of German adults aged 75 years and older [45] and a cross-sectional study of Greek adults with an average age of 75 years [46]. However, both studies used the EQ-5D [94] to assess HRQOL. Hajek et al. [45] also found a significant moderating effect of gender on the relationship between social support and HRQOL. In the complete case analysis, however, no association was found with physical HRQOL.

There were no significant associations between smoking and physical or mental HRQOL, even with a three-level (current smoker/former smoker/never smoker) or four-level (daily smoker/occasional smoker/former smoker/never smoker) categorisation. However, previous cross-sectional studies showed conflicting significant relationships. Amongst Brazilian adults aged 60 and older, current smokers reported worse quality of life than never smokers and former smokers [51]. In contrast, another study of Chinese adults aged 65 and above showed that current smokers had the highest HRQOL compared to never-smokers and former smokers [52]. In young Australian adults, smoking was cross-sectionally associated with lower physical HRQOL and longitudinally associated with reductions in physical HRQOL [95]. There are a lack of studies of older adults examining the association between smoking status and HRQOL. In particular, longitudinal analyses are necessary to provide evidence of causal relationships. Concerning alcohol consumption, we found a positive association only with physical HRQOL but not with mental HRQOL. Although the same cross-sectional results were detected in a study amongst Spanish adults aged 60 and older [53], prospective analysis could not confirm this association. Our results should be interpreted with caution as cross-sectional associations may reflect non-causal effects. Thus, we cannot assess whether older adults’ alcohol consumption may lead to better HRQOL or whether older adults who rate their HRQOL better tend to drink more alcohol. In a 14-year multi-wave study of Canadian adults aged 50 and older at baseline, moderate drinkers had the highest HRQOL compared to all other groups. Presumably, a worsening of health status is accompanied by a reduction in alcohol consumption, so the health benefits of alcohol consumption may be overestimated [54]. However, these findings need to be confirmed in further studies amongst older adult populations. As shown in previous studies [47, 48], physical activity was positively associated with physical and mental HRQOL.

As the explained variance of the model domains also depends on the order in which the factors are introduced, comparison with other studies seems difficult. It would be interesting for HRQOL research to examine how the contribution to the explained variance behaves in different countries and whether the contribution of the explained variance of the model domains differs compared to other age groups, such as adolescents or middle-aged adults.

### Strengths and limitations

The most important strength of this work is the large, up-to-date data set acquired through a whole population survey of older German adults that comprised a substantial variety of social and HRQOL factors. We assume that the full postal survey can yield high-quality data amongst older adults. In community-based settings, postal surveys provide clear advantages over telephone interviews and web surveys due to the availability of age-stratified postal addresses from the residents’ registration office. All returned questionnaires were filled out with great care, which may be attributed to specific characteristics of the age cohort and indicates high face validity. The comprehensive data allow a differentiated analysis of the associations between social factors and HRQOL for this specific target group. To the best of our knowledge, this is the first work to use comprehensive statistical models to investigate the relationships between sociodemographic, socioeconomic, psychosocial, and behavioural factors and both physical and mental HRQOL in older German adults. Using regression models, the associations of different social factors with physical and mental HRQOL can be weighed against one another, allowing more efficient and effective intervention targeting.

However, this work also has some limitations, so the presented findings should be interpreted with caution. Cross-sectional data sets are not suitable for determining causal relationships. Therefore, no causal inferences can be drawn from the results. For many reasons, self-reported data sets are inherently biased. Some subgroups are likely to be underrepresented in the data set, such as older adults living in residential care, older adults with lower SES, or older adults with specific disabilities or diseases. Compared to the population, there were fewer women (−3.5%), more adults aged 80 years and older (+ 5.7%), and fewer people with non-German citizenship (−4.7%) in our sample. Hence, we assume that the older adults in our sample were relatively privileged and healthy, which may underestimate associations between social factors and HRQOL in deprived subgroups. Data on HRQOL, alcohol consumption, and socioeconomic status could have been influenced by social desirability bias. Lower SES groups and older adults with disabilities or diseases may be more likely to be affected, resulting in an underestimation of associations. It should be considered that the impact of the COVID-19 pandemic may have worsened the HRQOL of older adults, which is likely to affect socially disadvantaged older adults in particular [17]*.* We assume specification errors in our empirical model affect model validity. Additional factors such as health complaints, chronic diseases, multi-morbidity, and disabilities as well as environmental factors may increase the goodness of fit of the models. Factors with low regression coefficients, such as smoking, partnership status, and occupational status, reduced the goodness of fit of the models. Presumably, introducing additional coping resources, such as trait resilience [96], sense of coherence [97], and self-efficacy, but also health literacy [98] would primarily increase the explained variance of mental HRQOL. The strengths and limitations of the data set have also been described in detail in another work by the authors [80].

### Implications for policy and practice

Particularly in ageing populations, reducing inequity in older adults’ HRQOL should be a primary aim of policy and practice. To date, the target groups who would most likely benefit from health interventions have often not been reached [99]. For example, groups with higher SES status tend to benefit disproportionately more from interventions than groups with lower SES status (inverse equity hypothesis), which can be explained by differences in accessibility, utilisation, and acceptance of interventions [100]. Given this phenomenon, the findings provide approaches for the development of tailored health interventions for older adults that can reach deprived target groups. To date, the relationships between social factors and HRQOL still seem to be underestimated in intervention development and implementation. From the results, risk profiles can be derived across target groups and fields of action, which holds considerable potential for interventions to maintain or improve HRQOL in older adults. In doing so, it may be adequate to address either physical or mental HRQOL specifically. Currently, there are promising efforts to target deprived older adults, e.g. female gender [101], low income [102], less physical activity [103], and less social support [104], for health interventions. Given that older adults with an external locus of control have lower SES and worse HRQOL, doctor-oriented communication of health recommendations may be beneficial to these populations. Age-specific offers, e.g. for adults aged 80 and over, could also be of assistance [105].

## Conclusion

In this analysis, sociodemographic, socioeconomic, psychosocial, and behavioural factors were associated with physical and mental HRQOL. These findings highlight the importance of social factors in older adults’ HRQOL and provide approaches for policy and practice to develop and implement tailored interventions amongst older adults to maintain or increase their HRQOL. We assume that our findings are transferable to municipalities in high-income countries in Europe. Our results provide a valuable contribution to HRQOL research in older adults. However, the social factors of HRQOL need to be further investigated in this specific age group. In the future work, we will analyse the relationship between social factors and HRQOL stratified by age, gender, and socioeconomic status.

## Data Availability

The data sets generated and/analysed during the current study are not publicly available due to restrictions imposed by the Puchheim municipal administration but are available from the corresponding author on reasonable request.

## References

[1] Hays RD, Morales LS (2001). The RAND-36 measure of health-related quality of life. Annals of Medicine.

[2] Machón M, Larrañaga I, Dorronsoro M, Vrotsou K, Vergara I (2017). Health-related quality of life and associated factors in functionally independent older people. BMC Geriatrics.

[3] Loayza LS, Valenzuela MT (2021). Health-related quality of life in older people with functional independence or mild dependence. Aging & Mental Health.

[4] Gunzelmann T, Albani C, Beutel M, Brähler E (2006). Subjective health of older people in view of the SF-36: Values from a large community-based sample [Subjective health of older people in view of the SF-36: Values from a large community-based sample]. Zeitschrift für Gerontologie und Geriatrie.

[5] Salive ME (2013). Multimorbidity in older adults. Epidemiologic Reviews.

[6] Zhang L, Ma L, Sun F, Tang Z, Chan P (2020). A multicenter study of multimorbidity in older adult inpatients in China. The Journal of Nutrition, Health & Aging.

[7] Ofori-Asenso R, Chin KL, Curtis AJ, Zomer E, Zoungas S, Liew D (2019). Recent patterns of multimorbidity among older adults in high-income countries. Population Health Management.

[8] Kshatri JS, Palo SK, Bhoi T, Barik SR, Pati S (2020). Prevalence and patterns of multimorbidity among rural elderly: Findings of the AHSETS study. Frontiers in Public Health.

[9] Okoro CA, Hollis ND, Cyrus AC, Griffin-Blake S (2018). Prevalence of disabilities and health care access by disability status and type among adults—United States, 2016. Morbidity and Mortality Weekly Report.

[10] Destatis. (2016). Older people in Germany and the EU. Wiesbaden, Germany.

[11] Andresen EM, Vahle VJ, Lollar D (2001). Proxy reliability: Health-related quality of life (HRQoL) measures for people with disability. Quality of Life Research.

[12] Albrecht GL, Devlieger PJ (1999). The disability paradox: High quality of life against all odds. Social Science & Medicine..

[13] Brown DS, Thompson WW, Zack MM, Arnold SE, Barile JP (2015). Associations between health-related quality of life and mortality in older adults. Prevention Science.

[14] Tsai S-Y, Chi L-Y, Lee C-H, Chou P (2007). Health-related quality of life as a predictor of mortality among community-dwelling older persons. European Journal of Epidemiology.

[15] Otero-Rodríguez A, León-Muñoz LM, Balboa-Castillo T, Banegas JR, Rodríguez-Artalejo F, Guallar-Castillón P (2010). Change in health-related quality of life as a predictor of mortality in the older adults. Quality of Life Research.

[16] Schlomann A, Bünning M, Hipp L, Wahl H-W (2022). Aging during COVID-19 in Germany: A longitudinal analysis of psychosocial adaptation. European Journal of Ageing.

[17] Horn V, Semmler M, Schweppe C (2023). Older people in Germany during the COVID-19 pandemic: The least, the more, and the most affected. Journal of Population Ageing.

[18] Brandl C, Zimmermann ME, Günther F, Dietl A, Küchenhoff H, Loss J, Stark KJ, Heid IM (2022). Changes in healthcare seeking and lifestyle in old aged individuals during COVID-19 lockdown in Germany: The population-based AugUR study. BMC Geriatrics.

[19] United Nations. (2020). World population ageing 2020: Highlights. New York.

[20] Commission E (2020). Ageing Europe: Looking at the lives of older people in the EU.

[21] Destatis. (2022). Elderly people: The population group of older people aged 65 and over. Wiesbaden, Germany.

[22] Wiles JL, Leibing A, Guberman N, Reeve J, Allen RES (2012). The meaning of “aging in place” to older people. The Gerontologist.

[23] Vanleerberghe P, de Witte N, Claes C, Schalock RL, Verté D (2017). The quality of life of older people aging in place: A literature review. Quality of Life Research.

[24] Cutchin MP (2003). The process of mediated aging-in-place: A theoretically and empirically based model. Social Science & Medicine.

[25] Davey JA, de Joux V, Nana G, Arcus M (2004). Accommodation options for older people in Aotearoa/New Zealand.

[26] Bao X-Y, Xie Y-X, Zhang X-X, Peng X, Huang J-X, Du Q-F, Wang P-X (2019). The association between multimorbidity and health-related quality of life: A cross-sectional survey among community middle-aged and elderly residents in southern China. Health and Quality of Life Outcomes.

[27] Wikman A, Wardle J, Steptoe A (2011). Quality of life and affective well-being in middle-aged and older people with chronic medical illnesses: A cross-sectional population based study. PLoS ONE.

[28] Makovski TT, Schmitz S, Zeegers MP, Stranges S, van den Akker M (2019). Multimorbidity and quality of life: Systematic literature review and meta-analysis. Ageing Research Review.

[29] Chang N-T, Chi L-Y, Yang N-P, Chou P (2010). The impact of falls and fear of falling on health-related quality of life in Taiwanese elderly. Journal of Community Health Nursing.

[30] Sunde S, Hesseberg K, Skelton DA, Ranhoff AH, Pripp AH, Aarønæs M, Brovold T (2021). Associations between health-related quality of life and physical function in older adults with or at risk of mobility disability after discharge from the hospital. Eur Geriatr Med..

[31] Sivertsen H, Bjørkløf GH, Engedal K, Selbæk G, Helvik A-S (2015). Depression and quality of life in older persons: A review. Dementia and Geriatric Cognitive Disorders.

[32] Porensky EK, Dew MA, Karp JF, Skidmore E, Rollman BL, Shear MK, Lenze EJ (2009). The burden of late-life generalized anxiety disorder: Effects on disability, health-related quality of life, and healthcare utilization. The American Journal of Geriatric Psychiatry.

[33] Yun J, Lee Y, Lee H-J (2022). A comparison of health-related quality of life and personal, social, and environmental factors of older adults according to a residential area: A propensity score matching analysis. Quality of Life Research.

[34] Gu Y, Zhang H, Ali SH, Huang M, Wei J, Gu S, Zhen X, Hu X, Sun X, Dong H (2019). Social determinants of health-related quality of life among residents in Zhejiang and Qinghai, China. International Journal of Environmental Research and Public Health.

[35] Tajvar M, Arab M, Montazeri A (2008). Determinants of health-related quality of life in elderly in Tehran, Iran. BMC Public Health.

[36] Boehlen FH, Maatouk I, Friederich H-C, Schoettker B, Brenner H, Wild B (2021). Loneliness as a gender-specific predictor of physical and mental health-related quality of life in older adults. Quality of Life Research.

[37] König H-H, Heider D, Lehnert T, Riedel-Heller SG, Angermeyer MC, Matschinger H, Villagut G, Bruffaerts R, Haro JM, de Giranolo G, de Graaf R, Kovess V, Alonso J, the ESEMeD/MHEDEA 2000 investigators (2010). Health status of the advanced elderly in six European countries: Results from a representative survey using EQ-5D and SF-12. Health and Quality of Life Outcomes.

[38] Abolhassani N, Santos-Eggimann B, Bula C, Goy R, Guessous I, Henchoz Y (2019). Temporal changes in importance of quality of life domains: A longitudinal study in community-dwelling Swiss older people. Quality of Life Research.

[39] Ma X, McGhee SM (2013). A cross-sectional study on socioeconomic status and health-related quality of life among elderly Chinese. British Medical Journal Open.

[40] Chen Y, Hicks A, While AE (2014). Quality of life and related factors: A questionnaire survey of older people living alone in Mainland China. Quality of Life Research.

[41] Jalenques I, Rondepierre F, Rachez C, Lauron S, Guiguet-Auclair C (2020). Health-related quality of life among community-dwelling people aged 80 years and over: A cross-sectional study in France. Health and Quality of Life Outcomes.

[42] Malicka B, Skośkiewicz-Malinowska K, Kaczmarek U (2022). The impact of socioeconomic status, general health and oral health on health-related quality of life, oral health-related quality of life and mental health among polish older adults. BMC Geriatrics.

[43] Helvik A-S, Bjørkløf GH, Corazzini K, Selbæk G, Laks J, Østbye T, Engedal K (2016). Are coping strategies and locus of control orientation associated with health-related quality of life in older adults with and without depression?. Archives of Gerontology and Geriatrics.

[44] Kostka T, Jachimowicz V (2010). Relationship of quality of life to dispositional optimism, health locus of control and self-efficacy in older subjects living in different environments. Quality of Life Research.

[45] Hajek A, Brettschneider C, Lange C, Posselt T, Wiese B, Steinmann S, Weyerer S, Werle J, Pentzek M, Fuchs A, Stein J, Luck T, Bickel H, Mösch E, Wolfsgruber S, Heser K, Maier W, Scherer M, Riedel-Heller SG, König HH (2016). Gender differences in the effect of social support on health-related quality of life: Results of a population-based prospective cohort study in old age in Germany. Quality of Life Research.

[46] Sarla E, Lambrinou E, Galanis P, Kalokairinou A, Sourtzi P (2020). Factors that influence the relationship between social support and health-related quality of life of older people living in the community. Gerontology and Geriatric Medicine.

[47] Zhang X, Tan SS, Franse CB, Alhambra-Borrás T, Verma A, Williams G, van Grieken A, Raat H (2021). Longitudinal association between physical activity and health-related quality of life among community-dwelling older adults: A longitudinal study of Urban Health Centres Europe (UHCE). BMC Geriatrics.

[48] Halaweh H, Willen C, Grimby-Ekman A, Svantesson U (2015). Physical activity and health-related quality of life among community dwelling elderly. Journal of Clinical Medical Research.

[49] Tseng H-Y, Löckenhoff C, Lee C-Y, Yu S-H, Wu I-C, Chang H-Y, Chiu Y-F, Hsiung CA (2020). The paradox of aging and health-related quality of life in Asian Chinese: Results from the healthy aging longitudinal study in Taiwan. BMC Geriatrics.

[50] Kennair LEO, Hagen R, Hjemdal O, Havnen A, Ryum T, Solem S (2022). Depression, anxiety, insomnia, and quality of life in a representative community sample of older adults living at home. Frontiers in Psychology.

[51] Viana DA, Andrade FCD, Martins LC, Rodrigues LR, Dos Santos Tavares DM (2019). Differences in quality of life among older adults in Brazil according to smoking status and nicotine dependence. Health and Quality of Life Outcomes.

[52] Jing Z, Li J, Wang Y, Yuan Y, Zhao D, Hao W, Yu C, Zhou C (2021). Association of smoking status and health-related quality of life: Difference among young, middle-aged, and older adults in Shandong, China. Quality of Life Research.

[53] Ortolá R, García-Esquinas E, Galán I, Rodríguez-Artalejo F (2016). Patterns of alcohol consumption and health-related quality of life in older adults. Drug and Alcohol Dependence.

[54] Kaplan MS, Huguet N, Feeny D, McFarland BH, Caetano R, Bernier J, Giesbrecht N, Oliver L, Ross N (2012). Alcohol use patterns and trajectories of health-related quality of life in middle-aged and older adults: A 14-year population-based study. Journal of Studies on Alcohol and Drugs.

[55] Janßen C, Sauter S, Kowalski C (2012). The influence of social determinants on the use of prevention and health promotion services: Results of a systematic literature review. GMS Psycho-Social Medicine..

[56] Mackenbach JP (2006). Health inequalities: Europe in profile.

[57] Dahlgren G, Whitehead M (1991). Policies and strategies to promote social equity in health: Background document to WHO—Strategy paper for Europe.

[58] Ware JE, Sherbourne CD (1992). The MOS 36-ltem short-form health survey (SF-36). Medical Care..

[59] Ware JE, Kosinski M, Bjorner JB, Turner-Bowker DM, Gandek B, Maruish ME (2007). User’s manual for the SF-36v2 health survey.

[60] Ellert U, Kurth BM (2013). Health related quality of life in adults in Germany: Results of the German health interview and examination survey for adults (DEGS1). Bundesgesundheitsblatt, Gesundheitsforschung, Gesundheitsschutz.

[61] Bellach B-M, Ellert U, Radoschewski M (2000). The application of the short form questionnaire 36 within the national health survey—First results and new questions. Bundesgesundheitsblatt—Gesundheitsforschung—Gesundheitsschutz..

[62] Morfeld M, Bullinger M, Nantke J, Brähler E (2005). The version 2.0 of the SF-36 health survey: Results of a population-representative study. Sozial- und Präventivmedizin.

[63] Hayes V, Morris J, Wolfe C, Morgan M (1995). The SF-36 health survey questionnaire: Is it suitable for use with older adults?. Age and Ageing.

[64] Walters SJ, Munro JF, Brazier JE (2001). Using the SF-36 with older adults: A cross-sectional community-based survey. Age and Ageing.

[65] Bjerk M, Brovold T, Skelton DA, Bergland A (2018). Associations between health-related quality of life, physical function and fear of falling in older fallers receiving home care. BMC Geriatrics.

[66] Kaiser HF (1974). An index of factorial simplicity. Psychometrika.

[67] Field AP (2018). Discovering statistics using IBM SPSS statistics.

[68] Lampert T, Kroll LE, Müters S, Stolzenberg H (2013). Measurement of the socioeconomic status within the German Health Update 2009 (GEDA). Bundesgesundheitsblatt, Gesundheitsforschung, Gesundheitsschutz.

[69] Lampert T, Müters S, Stolzenberg H, Kroll LE (2014). Measurement of socioeconomic status in the KiGGS study: First follow-up (KiGGS wave 1). Bundesgesundheitsblatt, Gesundheitsforschung, Gesundheitsschutz.

[70] Lampert T, Hoebel J, Kuntz B, Müters S, Kroll LE (2018). Socioeconomic status and subjective social status measurement in KiGGS Wave 2. Journal of Health Monitoring.

[71] von Lengerke T, Janssen C, John J (2007). Sense of coherence, health locus of control, and quality of life in obese adults: Physical limitations and psychological normalcies. International Journal of Public Health.

[72] Dalgard OS, Bjørk S, Tambs K (1995). Social support, negative life events and mental health. British Journal of Psychiatry.

[73] Kilpeläinen K, Aromaa A (2008). European health indicators: Development and initial implementation: Final report of the ECHIM project.

[74] Bush K, Kivlahan DR, McDonell MB, Fihn SD, Bradley KA (1998). The AUDIT alcohol consumption questions (AUDIT-C): An effective brief screening test for problem drinking: Ambulatory care quality improvement project (ACQUIP): Alcohol use disorders identification test. Archives of Internal Medicine.

[75] Wohlrab D (2018). Schwerpunktbefragung der Münchner Bürgerinnen und Bürger zur sozialen und gesundheitlichen Lage 2016: Ausgewählte Aspekte zur Lage von gesundheitlich beeinträchtigten Zielgruppen.

[76] Zeiher J, Kuntz B, Lange C (2017). Smoking among adults in Germany. Journal of Health Monitoring.

[77] Lindqvist A, Sendén MG, Renström EA (2021). What is gender, anyway: A review of the options for operationalising gender. Psychology & Sexuality..

[78] Eagly AH (2004). Psychology of gender.

[79] Beckmann K, Glemser A, Heckel C, Heyde C, von der Hoffmeyer-Zlotnik JH, Hanefeld U, Herter-Eschweiler R, Kühnen C (2016). Demographische Standards: Eine gemeinsame Empfehlung des ADM, Arbeitskreis Deutscher Markt- und Sozialforschungsinstitute e.V., der Arbeitsgemeinschaft Sozialwissenschaftlicher Institute e.V. (ASI) und des Statistischen Bundesamtes.

[80] Geigl C, Loss J, Leitzmann M, Janssen C (2022). Social factors of dietary risk behavior in older German adults: Results of a multivariable analysis. Nutrients.

[81] Rubin DB (1987). Multiple imputation for nonresponse in surveys.

[82] Little RJA, Rubin DB (2002). Statistical analysis with missing data.

[83] Tilling K, Williamson EJ, Spratt M, Sterne JAC, Carpenter JR (2016). Appropriate inclusion of interactions was needed to avoid bias in multiple imputation. Journal of Clinical Epidemiology.

[84] Pedersen AB, Mikkelsen EM, Cronin-Fenton D, Kristensen NR, Pham TM, Pedersen L, Petersen I (2017). Missing data and multiple imputation in clinical epidemiological research. Clinical Epidemiology.

[85] Vink G, Frank LE, Pannekoek J, van Buuren S (2014). Predictive mean matching imputation of semicontinuous variables. Statistica Neerlandica.

[86] Jakobsen JC, Gluud C, Wetterslev J, Winkel P (2017). When and how should multiple imputation be used for handling missing data in randomised clinical trials—A practical guide with flowcharts. BMC Medical Research Methodology.

[87] Cohen J (1988). Statistical power analysis for the behavioral sciences.

[88] Urban D, Mayerl J (2018). Angewandte Regressionsanalyse: Theorie, Technik und Praxis.

[89] van Ginkel JR (2019). Significance tests and estimates for r2 for multiple regression in multiply imputed datasets: A cautionary note on earlier findings, and alternative solutions. Multivariate Behavioral Research.

[90] Hayes AF, Cai L (2007). Using heteroskedasticity-consistent standard error estimators in OLS regression: An introduction and software implementation. Behavior Research Methods.

[91] Wallston KA, Wallston BS, DeVellis R (1978). Development of the multidimensional health locus of control (MHLC) scales. Health Education Monographs.

[92] Rotter JB (1954). Social learning and clinical psychology.

[93] Geigl C, Spagert L, Loss J, Leitzmann M, Janssen C (2022). Older German adults’ health-related quality of life and associated social factors. European Journal of Public Health, 32(Supplement_3).

[94] Rabin R, de Charro F (2001). EQ-5D: A measure of health status from the EuroQol group. Annals of Medicine.

[95] Tian J, Venn AJ, Blizzard L, Patton GC, Dwyer T, Gall SL (2016). Smoking status and health-related quality of life: A longitudinal study in young adults. Quality of Life Research.

[96] Färber F, Rosendahl J (2020). Trait resilience and mental health in older adults: A meta-analytic review. Personality and Mental Health.

[97] Drageset J, Nygaard HA, Eide GE, Bondevik M, Nortvedt MW, Natvig GK (2008). Sense of coherence as a resource in relation to health-related quality of life among mentally intact nursing home residents—a questionnaire study. Health and Quality of Life Outcomes.

[98] Lee MK, Oh J (2020). Health-related quality of life in older adults: Its association with health literacy, self-efficacy, social support, and health-promoting behavior. Healthcare.

[99] Liljas AEM, Walters K, Jovicic A, Iliffe S, Manthorpe J, Goodman C, Kharicha K (2019). Engaging ‘hard to reach’ groups in health promotion: The views of older people and professionals from a qualitative study in England. BMC Public Health.

[100] Mackenbach JP (2012). The persistence of health inequalities in modern welfare states: The explanation of a paradox. Social Science and Medicine.

[101] Lawlor ER, Cupples ME, Donnelly M, Tully MA (2019). Promoting physical activity among community groups of older women in socio-economically disadvantaged areas: Randomised feasibility study. Trials.

[102] Geffen LN, Kelly G, Morris JN, Howard EP (2019). Peer-to-peer support model to improve quality of life among highly vulnerable, low-income older adults in Cape Town, South Africa. BMC Geriatrics.

[103] Kwan RYC, Salihu D, Lee PH, Tse M, Cheung DSK, Roopsawang I, Choi KS (2020). The effect of e-health interventions promoting physical activity in older people: A systematic review and meta-analysis. European Review of Aging and Physical Activity.

[104] Hirani SS, Norris CM, van Vliet KJ, van Zanten SV, Karmaliani R, Lasiuk G (2018). Social support intervention to promote resilience and quality of life in women living in Karachi, Pakistan: A randomized controlled trial. International Journal of Public Health.

[105] Gustafsson S, Wilhelmson K, Eklund K, Gosman-Hedström G, Zidén L, Kronlöf GH, Højgaard B, Slinde F, Rothenberg E, Landahl S, Dahlin-Ivanoff S (2012). Health-promoting interventions for persons aged 80 and older are successful in the short term–results from the randomized and three-armed elderly persons in the risk zone study. Journal of the American Geriatrics Society.

